# Supplementary material for: Molecular embroidering of graphene
Source: Nat Commun. 2021 Jan 22;12:552. doi: 10.1038/s41467-020-20651-w (PMC7822905; doi:10.1038/s41467-020-20651-w)
Supplement: Supplementary file 1 — Supplementary Information [file 41467_2020_20651_MOESM1_ESM.pdf]

Supplementary Information

Molecular embroidering of graphene

Tao Wei, Malte Kohring, Heiko B. Weber, Frank Hauke, and Andreas Hirsch*

Content

S1. Materials

S2. Patterned graphene fabrication by EBL

S3. Step-wise covalent functionalization of graphene in the respective patterned areas

S4. Raman characterization of functionalized areas: Reduction time and degree of functionalization

S5. Reference experiments: Functionalization of non-activated graphene areas

S6. Reference experiments: Re-reduction of covalently functionalized graphene areas

S1. Materials

CVD graphene on a 1×1 cm^2 polymethyl methacrylate (PMMA) substrate was purchased from ACS Material Co. (USA). All other chemicals were purchased from Sigma Aldrich Co. (Germany). Iodine monochloride (ICl) and ethanol as solvent were dried for three days over 4 Å molecular sieves, which were preheated under vacuum for another three days. Subsequently, the dried ICl, ethanol, and the deuteroxide were degassed by pump freeze (seven iterative steps) and transferred to an argon filled glovebox (< 0.1 ppm O_2, < 0.1 ppm H_2O).

S2. Patterned graphene fabrication by EBL

The graphene monolayer was deposited on a Si/SiO_2 wafer by a wet transfer technique. Here, the PMMA-supported graphene, floating on top of a water surface, was fished onto the prepared Si/SiO_2 wafer. Subsequently, the PMMA coating was removed by acetone vapor (60 min) and the wafer was dried in air. Afterwards, a fresh PMMA double-layer mask was applied by spin coating (3,500 rpm, 35 s) a 200 nm thick layer of PMMA 200 A7 (dissolved in anisole, solid content: 7%) and subsequently a 200 nm thick layer of PMMA 950 A4 (dissolved in anisole, solid content: 4%), followed by a bake step for each applied layer in air (layer 1: 180 °C, 60 s; layer 2: 180 °C, 90 s).

The circular patterns in the PMMA mask (array of 40 x 40 circles) for the subsequent three reductive functionalization sequences were generated *via* electron-beam lithography (EBL). The respective patterns are generated on a Zeiss Supra SEM (10 kV). With this low acceleration voltage, radiation-related defects in the graphene layer can widely be avoided.[S1] Irradiated PMMA areas were cleaned by washing the wafer with an isopropanol/methyl isobutyl ketone (3:1) solution. After the exposure of the free graphene surface in the respective patterned areas, the reductive activation/functionalization protocol has been carried out (see section S3). The layout of the three different pattern areas (A, B, and C) are summarized in Supplementary Figure 1.

After each individual functionalization step, the complete PMMA mask (which becomes partly damaged by the addition of the electron-trapping reagent benzonitrile, see section S3) was completely removed by acetone vapor (60 min) and a fresh PMMA double-layer mask has been applied *via* spin-coating to enable the next EBL patterning cycle. In this case, the previously created addend patterns become covered by the freshly deposited PMMA layer and thus the localization of the chemically functionalized areas turns out to be tricky. In order to determine the exact positions of the covalently functionalized patterned areas we initially applied a crosshair pattern (with additional letters for a better orientation, Supplementary Figure 1) on the graphene by an e-beam lithography procedure with a subsequent 5 nm titanium and 40 nm gold evaporation step (5,000 µm x 5,000 µm write field and an aperture of 120 µm

with a dose of 200 μAs cm^{-2}). Based on these set of distinct markers, the position for the subsequent EBL patterning spots can be located and the respective concentric shapes can be "written".

In the first EBL patterning step, periodic dots with a hole diameter of 5 μm were generated. The subsequent reductive arylation reaction has been carried out as described in section S3.

In the second EBL patterning step, periodic dots with a hole diameter of 15 μm were "written" into the PMMA mask. As it is demonstrated by the SEM-EDS analyses of the final multi-functionalized sample (Figure 5 in the main manuscript) this lithographic removal of the PMMA protective layer does not lead to a removal of the 4-bromobenze addends introduced in the first reductive activation/functionalization step. The subsequent reductive deuteration reaction has been carried out as described in section S3.

In the third patterning step the protective PMMA layer was removed in a ring type fashion around the initially arylated and deuterated areas (outer diameter 20 μm, inner diameter 15 μm). With this setup, the covalently functionalized zones I_b and II_b (diameter of 15 μm) remain covered by the protective PMMA layer. This approach has been chosen to reduce the possibility of a reduction based addend removal of the initially bound functional groups. As outlined and discussed in detail in section S6 – the addition of sodium/potassium (Na/K) alloy to the aryl bound functionalization zone I_b leads to a pronounced removal of bound entities upon re-reduction on long time scales (Supplementary Figure 8 and Figure 11). The subsequent reductive chlorination reaction has been carried out as described in section S3.

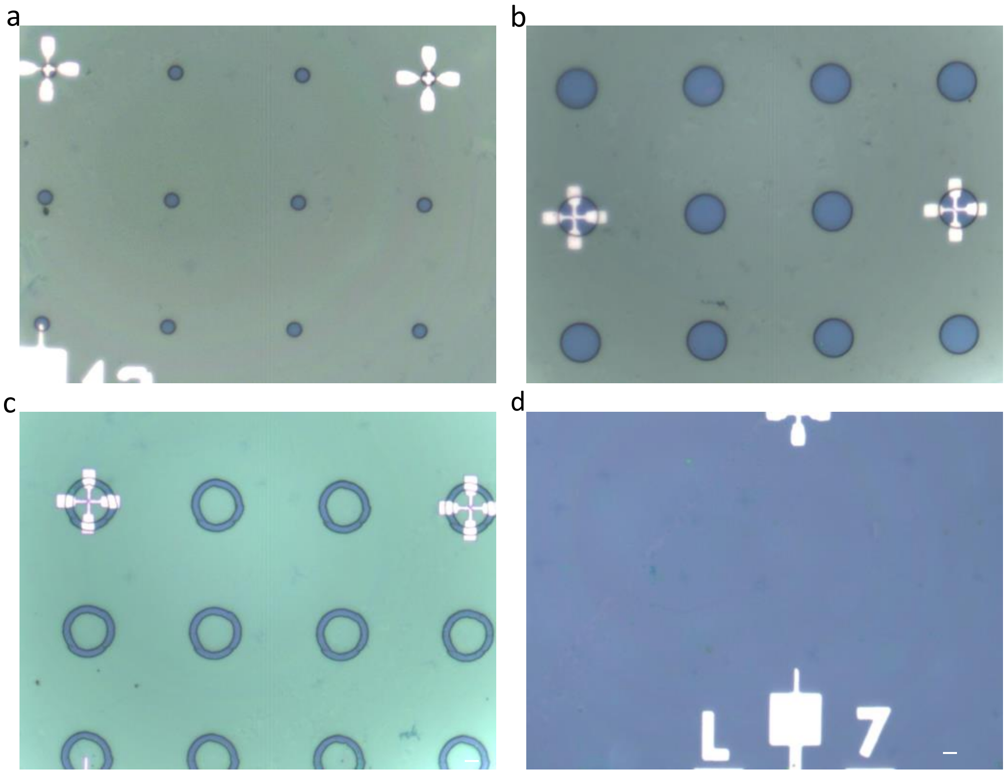

Supplementary Figure 1. Optical images of three iteratively patterned graphene areas (in total: array of 40 x 40 circles). (a) hole with 5 µm diameter, (b) holes with 15 µm, (c) concentric rings (outer diameter 20 µm, inner diameter 15 µm) – blueish regions represent the areas where the PMMA (polymethyl methacrylate) double layer (green) has been removed by EBL (electron-beam lithography). The lithographically applied crosshair pattern is used for the localization of the respective zones of functionalization after each covalent functionalization/PMMA-coating cycle. (d) Optical image of the final sample after threefold patterned functionalization and PMMA removal. Scale bar: 5 µm.

S3. Step-wise covalent functionalization of graphene in the respective patterned areas

In an argon filled glovebox (< 0.1 ppm O_2, < 0.1 ppm H_2O), the EBL-patterned graphene was initially activated by a reduction with a Na/K (molar ratio 1:3) alloy. Specifically, a drop of liquid Na/K alloy was dropped onto the surface of the sample and kept for 90 minutes – our reduction time based activation/functionalization study (see section S4) clearly shows a maximum for the final degree of functionalization after an activation time of 90 min (Raman spectra for each functionalization reagent are presented in Supplementary Figure 2, Figure3, and Figure 4, respectively) – leading to an efficient reductive activation of the exposed graphene areas.

This activation procedure, by reducing graphene directly with the liquid Na/K alloy, differs considerably from our previously developed method employing a Na/K-DME (DME = dimehtoxyethane) solution.[S2] The usage of DME would lead to a damaging of the protective PMMA double-layer and would render the whole area-restricted functionalization approach impossible.

After removal of the residual Na/K alloy by a constant flow of argon, several drops of 4-bromobenzenediazonium tetrafluoroborate dissolved in dried and degassed ethanol (0.5 mmol mL^{-1}) were intermittently added for 15 min. Afterwards, several drops of ethanol were added to remove the unreacted diazonium salt and then one drop of benzonitrile was added to terminate the reaction. As we have shown previously, benzonitrile is capable to remove residual negative charges on graphene.[S3] Subsequently, the sample was exported from the glovebox and washed with additional 20 mL ethanol and 20 mL water. Finally, the PMMA layer was removed by acetone vapor (60 min) and the sample was characterized spectroscopically.

For the second functionalization step, the arylated graphene sample was applied with a PMMA double-layer mask and patterned *via* EBL (see section S2), generating the second pattern structure. After undergoing reduction under the same condition as well as alloy removal, one drop of deuteroxide was added for 15 min for the second covalent functionalization reaction. Afterwards, the reaction was terminated by adding a drop of benzonitrile to quench the residual negative charges and washed with deuteroxide for several times. Again, the PMMA layer was removed by acetone vapor (60 min) and the

sample was characterized spectroscopically.

Finally, the third pattern of the concentric ring structure was created by EBL in analogy to the other two patterns. Following the strategy of reductive activation, a few drops of iodine monochloride (ICl) diluted with dried and degassed ethanol (0.5 mmol mL^{-1}) were intermittently added for 15 min. Afterwards, several drops of ethanol were added to remove the unreacted iodine monochloride and then one drop of benzonitrile was added to terminate the reaction. Subsequently, the sample was washed three times with 20 mL ethanol. Finally, acetone vapor (60 min) was used to remove the protective PMMA layer to give rise to the final sample of multiple-patterned functionalized graphene.

S4. Raman characterization of functionalized areas: Reduction time and degree of functionalization

Raman spectroscopy and in particular Scanning Raman Spectroscopy (SRS) represents a very powerful tool for the investigation of covalently functionalized graphene. This technique was therefore applied to characterize the multiply 2D-patterned sheet architectures. As shown in Supplementary Figure 2, Figure3, and Figure4 with increasing reduction time, the intensity of the D-band for addend zones I_b, II_b, and III_b increased progressively, suggesting the gradually improved degree of functionalization. Finally, the average I_D/I_G ratio of zones I_b, II_b, and III_b can be enhanced to about 2.6/1.1/2.4, respectively (Supplementary Figure 5 and Supplementary Table 1). It has to be pointed out that the reduction reached a maximum after 90 min as demonstrated by the unchanged I_D/I_G ratios. Remarkably, different from the arylation and chlorination (zones I_b and III_b), the deuteration (zone II_b) can achieve very high degrees of functionalization (located in the high-functionalization-regime of Cançado curve[S4]) as indicated by the very broad Raman D-band.

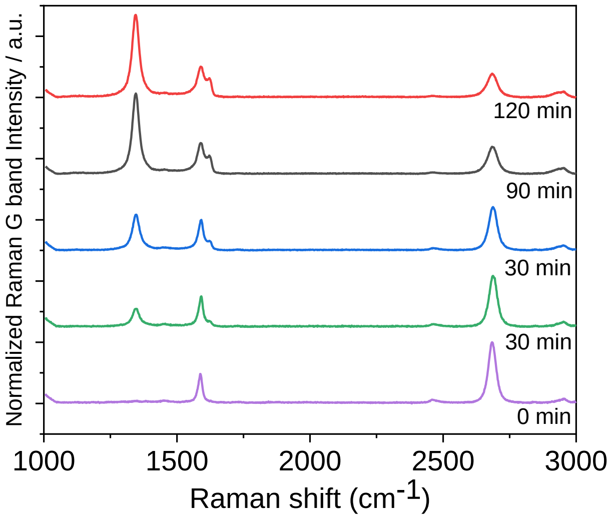

Supplementary Figure 2. Raman spectra for the reductive arylation of graphene within zone I_b. The different reduction time ranging from 0 min to 120 min are performed.

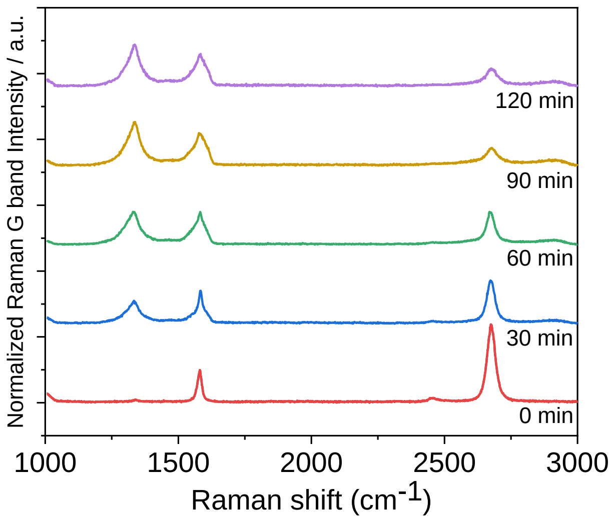

Supplementary Figure 3. Raman spectra for the reductive deuteration of graphene within zone II$_b$. The different reduction time ranging from 0 min to 120 min are performed.

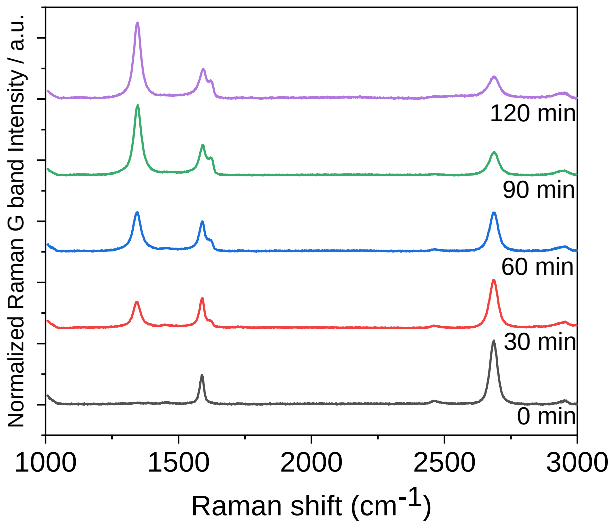

Supplementary Figure 4. Raman spectra for the reductive chlorination of graphene within zone III$_b$. The different reduction time ranging from 0 min to 120 min are performed..

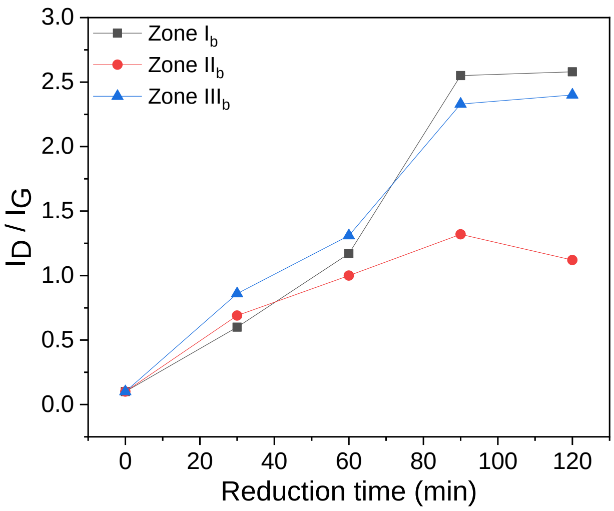

Supplementary Figure 5. Raman I_D/I_G ratios upon different reduction times for zone I_b, II_b, and III_b. I_D/I_G: Raman D band to G band intensity ratio

Supplementary Table 1: Raman data of different addend zones (I_b, II_b, and III_b) upon different reduction times, λ_{exc} = 532 nm.

Reduction Time / min	I_D/I_G for zoneI_b (Arylation)	I_D/I_G for zone II_b (Deuteration)	I_D/I_G for Region III_b (Chlorination)
0	<0.1	<0.1	<0.1
30	0.60	0.69	0.86
60	1.17	1.00	1.31
90	2.55	1.32	2.33
120	2.58	1.12	2.40

The key advantage of a reductive activation of graphene is that it can achieve a high degree of functionalization in the bulk material. We have also clearly demonstrated that this approach can be transferred from the bulk material to a single layer of graphene and provides a method to covalently functionalize mono-layer graphene in highly homogenous fashion.[S2] Besides the actual degree of functionalization, the homogeneity of the addend coverage represents another highly important factor. To further confirm this, we also carried out a reference experiment that a large size of graphene (0.5 × 0.5 cm) was deuterated upon reductive activation method and characterized by Raman spectroscopy (Supplementary Figure 6). It can be clearly seen that, compared to the spectral information presented for the patterned functionalization in Supplementary Figure 3 (after 90 min reduction time), a similar Raman features involving very broad Raman D, G bands together with I_D/I_G of 1.14 were observed upon reduction for 50 min. This indicates that the degree of functionalization (deuteration) is comparable with that of deuteration of micro-sized graphene (zone II_b) as expected and can be interpreted as a high degree of deuteration. Compared to patterned deuteration of graphene, the shorter reduction time (for unpatterned deuteration) can be explained by the easier reduction of graphene without PMMA covering.

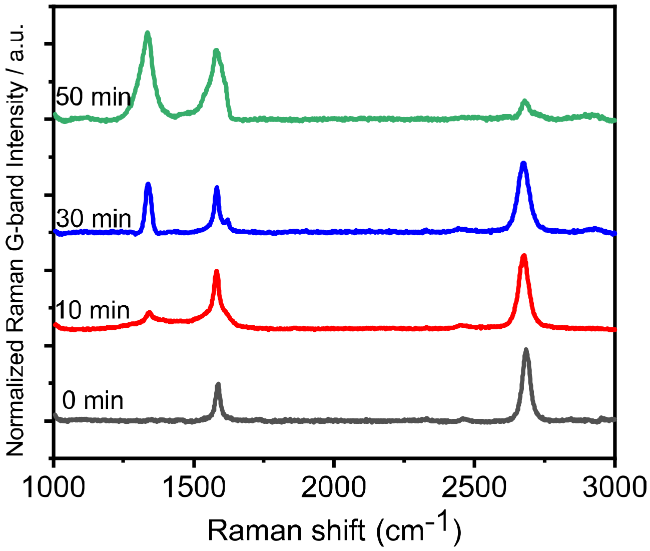

Supplementary Figure 6. Raman spectra for the reductive deuteration of large-scale graphene (0.5 × 0.5 cm) upon different reduction times. The reduction time ranging from 0 min to 50 min are performed.

The different electronic dispersion behaviors render the Raman spectra for single layer graphene and multilayer (or bulk) graphene completely different. Generally, the G' peak of single-layer graphene is sharp and symmetrical, and has a perfect single Lorentzien peak shape. However, as the number of graphene layers increases, the electronic energy band structure of graphene is split, thus leading to many possible double resonance scattering processes. This means the G' peak can be fitted into several Lorentzien peaks and the intensity of G peak also increases because more carbon atoms will be detected in the multilayer (or bulk) graphene. In view of this, the Raman spectroscopy has the advantage to clearly, efficiently and non-destructively characterize the monolayer-graphene and gives the intrinsic information of graphene. This also enables us to use Raman spectroscopy to clearly and easily quantify the degree of functionalization of monolayer graphene. In general, the Raman I_D/I_G ratios correlate with a mean defect distance L_D and the maximum I_D/I_G ratio corresponds to the L_D-crit value, which was used to distinguish the low-functionalization-regime and high-functionalization-regime.[S4,S5] To find out which regime the I_D/I_G ratio is located at, the width of Raman bands should be analyzed. Generally, on basis of the I_D/I_G ratio together with the full width at half maximum (FWHM) the degree of functionalization of graphene can be quantified. The observed FWHM values (< 30 cm^{-1}) of Raman spectra of zone I_b and III_b, indicate their location in the low-functionalization-regime and the L_D was calculated to be 6.3 nm and 6.6 nm, respectively. According to our previously introduced method, the degree of functionalization for zone I_b and III_b can be quantified as 0.059% and 0.053%.[S6] In contrast, the zone II_b can be assigned to the high-functionalization-regime – based on its very broad D-band with FWHM values > 30 cm^{-1}. Here, the degree of functionalization can be quantified to be 1.176%. Since the degree of functionalization of graphene based on our reductive activation strategy does not change upon varying the size of graphene, it can be reasonably compared with previous examples of graphene

patterning regardless of the difference between their sizes. To this end, we have also quantified previously reported cases on covalent patterning of monolayer graphene[S7-S10] for comparison (Supplementary Table 2) and here it becomes obvious that our iterative graphene functionalization protocol leads to a highly pronounced functionalization of the respective areas.

Supplementary Table 2: Comparison of quantified degree of functionalization (*θ*) of previously reported covalent patterning of graphene and this work.

	Ref S7	Ref S8	Ref S9	Ref S10	Zone I_b	Zone II_b	Zone III_b
I_D / I_G	0.25	0.56	0.72	0.80	2.58	1.12	2.40
θ %	0.004	0.009	0.012	0.014	0.059	1.176	0.053

S5. Reference experiments: Functionalization of non-activated graphene areas

In order to shed light on the initial reductive activation process, we also conducted specific reference experiments. Here, the EBL-patterned graphene – periodic dots of 10 µm diameter – was directly reacted with 4-bromobenzenediazonium tetrafluoroborate, D_2O, and ICl under identical conditions – but without the initial reductive activation step – and under analogue work-up/post functionalization treatment. The corresponding Raman spectra are shown in Supplementary Figure 7. It can be clearly seen that the Raman D-band for the two functionalization steps based on the reaction with D_2O and ICl do not exhibit an intensity increase in comparison to the starting material, suggesting that the reaction cannot occur without a reductive activation even though it is thinkable that the EBL treatment may also lead to a slight activation of the graphene surface in this respective areas. On the other hand, in the sample reacted with 4-bromobenzenediazonium tetrafluoroborate, a slight D-band increase to a I_D/I_G ratio of 0.13 (before reaction the $I_D/I_G < 0.1$) can be detected. – indicating a very low degree of functionalization compared with our reductive strategy (I_D/I_G ratio of 2.6).

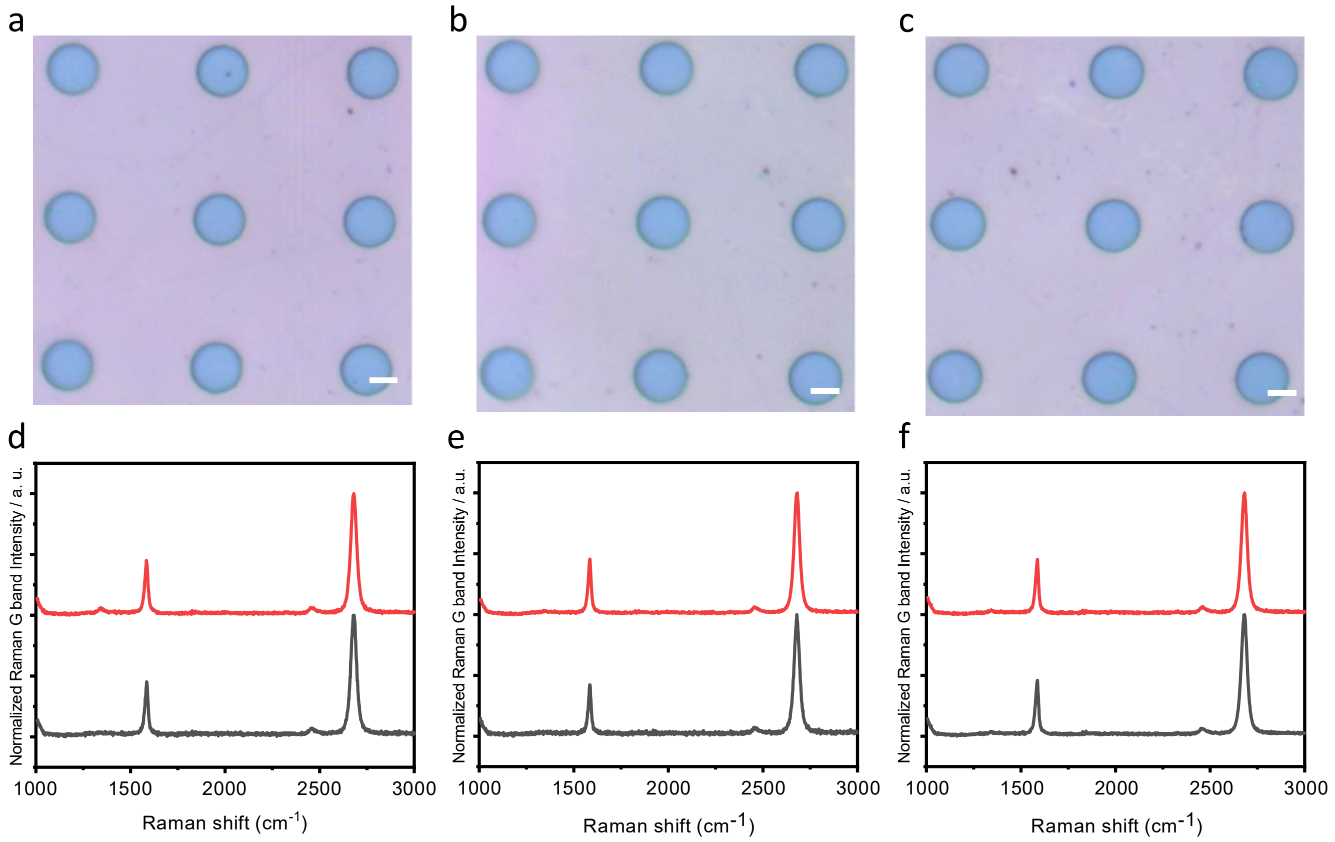

Supplementary Figure 7. Optical images of graphene patterns and the corresponding Raman spectra. (a, b, c) periodic dots of 10 μm diameter. The blue cyclic dots are areas where PMMA (polymethyl methacrylate, purple) has been removed by EBL (electron-beam lithography) such that the graphene become exposed. The Raman spectra of the EBL-exposed graphene zones before and after reaction (without initial reductive activation) with 4-bromobenzene diazonium-tetrafluoroborate (d), D_2O (e), and ICl (f). Scale bar: 5 μm.

S6. Reference experiments: Re-reduction of covalently functionalized graphene areas

In order to shed light in the possibility of an addend removal upon the addition of the reducing reagent[S2,S11] to covalently functionalized graphene areas we have treated the multiple-patterned functionalized graphene with Na/K alloy and tracked the respective spectroscopic Raman changes over time (Supplementary Figure 8-10).

Here it becomes apparent that the different addend zones (I_b, II_b, and III_b) equipped with differing molecular entities, covalently bound to the graphene lattice, exhibit different de-functionalization behaviors upon reduction. For zones I_b and III_b, as the reduction time increases, the Raman D-band decreases continuously, which is indicative for an ongoing de-functionalization reaction based on a cleavage of the respective addend-graphene bonds (Supplementary Figure 8, and Figure10). After 4 h of reductive treatment, the D-band intensity of the arylated graphene area (zone I_b) has almost vanished, resulting in a I_D/I_G ratio of around 0.2 (Supplementary Figure 11 and Table 3) and the Raman spectrum

resembles that of pristine graphene. On the other hand, short reductive treatment only leads to a minor decrease of the respective D-band intensity (Supplementary Figure 11). Our finding is in clear line with similar studies carried out for carbon nanotubes[S2], bulk graphene[S11] and C_{60} derivatives[S12,S13] and a clear indication that also in monolayer graphene systems, a post functionalization reduction leads to a detachment of the previously introduced covalent functionalities.

In analogy, the chlorinated graphene within zone III_b could also be transformed back to pristine graphene upon 3 h of reductive treatment – corroborated by a complete disappearance of the respective Raman D-band (Supplementary Figure 10 and Figure 11). Consequently, the I_D/I_G ratio is reduced to around 0.1 (Supplementary Table 3).

However, a completely different behavior was observed for the deuterated graphene within zone II_b (Supplementary Figure 9). After 4 h of reductive treatment, the corresponding Raman D-band remains unchanged. A subsequent extension of the re-reduction time to 8 h or even to 24 h does not lead to a detectable change of the Raman D-band intensity (Supplementary Figure 11 and Table 3), indicative for the high stability of the connected C-D bond. These results are in line with the thermal desorption investigations (as discussed in main text) and can be well explained considering the different bond energies for the graphene-lattice-sp^3/addend bond ($E_{C-D(H)}$ (414 kJ mol^{-1}) > E_{C-C} (332 kJ mol^{-1}) > E_{C-Cl} (328 kJ mol^{-1})) within the respective addend zones. The significantly higher bond energy of the C-D bond accounts for its high stability, in good agreement with the temperature-dependent Raman results.

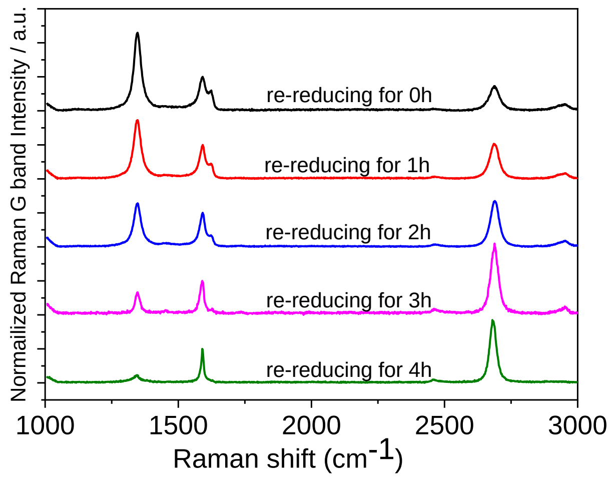

Supplementary Figure 8. Raman spectra of the de-arylation of graphene within zone I_b upon different re-reduction times. The re-reduction time ranging from 0 h to 4 h are performed.

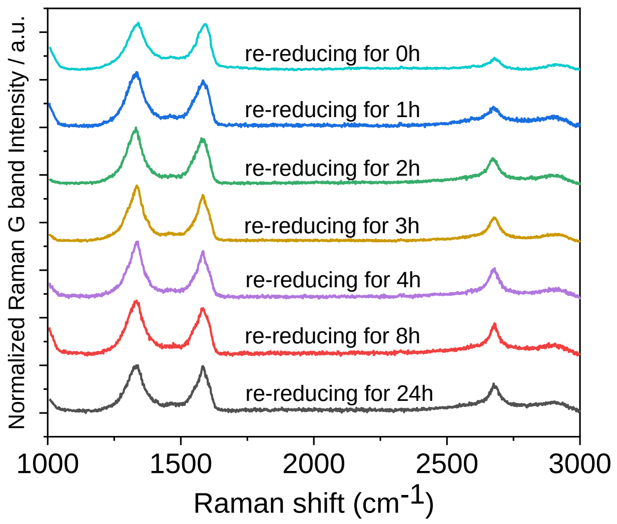

Supplementary Figure 9. Raman spectra of the behavior of zone II_b upon different re-reduction times. The re-reduction time ranging from 0 h to 24 h are performed.

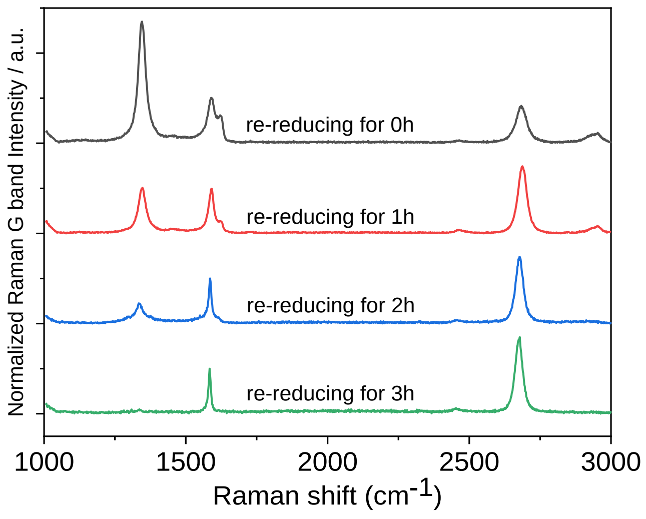

Supplementary Figure 10. Raman spectra of de-chlorination of graphene within zone III_b upon different re-reduction times. The re-reduction time ranging from 0 h to 3 h are performed.

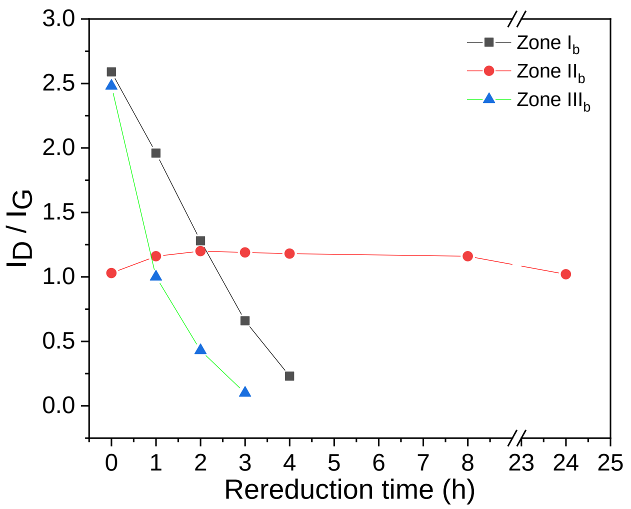

Supplementary Figure 11. Raman I_D/I_G ratios upon different re-reduction times for zone I_b, II_b, and III_b.

Supplementary Table 3: Raman data of different addend zones (I_b, II_b, and III_b) upon different re-reduction times, λ_{exc} = 532 nm.

Rereduction Time / h	I_D/I_G for zone I_b (Dearylation)	I_D/I_G for zone II_b (Dedeuteration)	I_D/I_G for zone III_b (Dechlorination)
0	2.59	1.03	2.48
1	1.96	1.16	1.00
2	1.28	1.20	0.43
3	0.66	1.19	0.10
4	0.23	1.18	/
8	/	1.16	/
24	/	1.02	/

Based on our results, the degree of functionalization in the different addend zones (I_b-III_b) can be fine-tuned by the treatment time of post-functionalization added Na/K alloy (Supplementary Scheme 1).

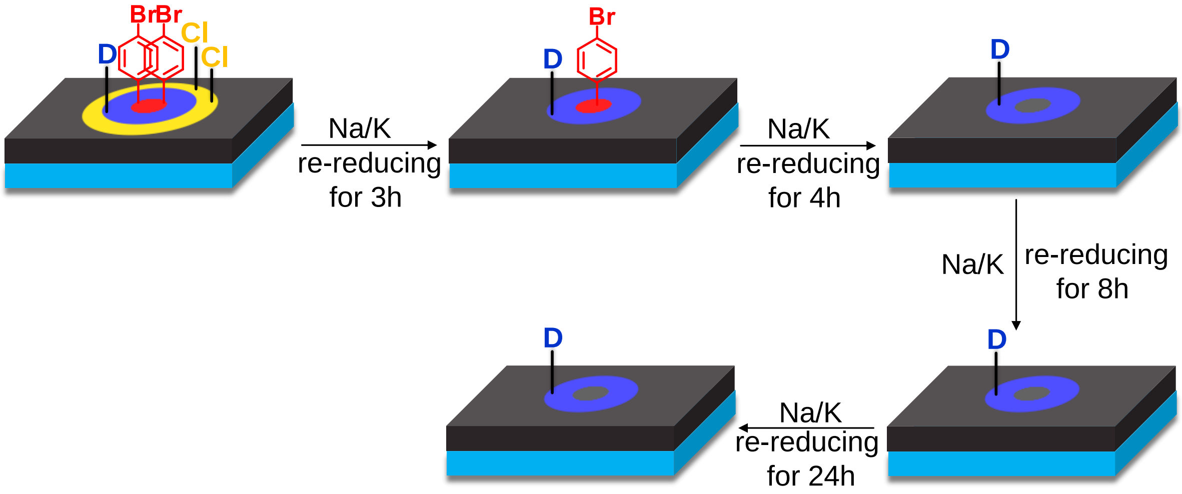

Supplementary Figure 12. Schematic illustration of de-functionalization upon re-reduction treatment by sodium/potassium (Na/K) alloy. Red circle indicates graphene regions bound with bromophenyl units. Blue concentric ring indicates graphene regions bound with deutero atoms. Orange concentric ring indicates graphene regions bound with chloro atoms.

References:

1. Meyer, J. C. *et al.*, "Accurate measurement of electron beam induced displacement cross sections for single-layer graphene", *Phys. Rev. Lett.* **108**, 196102-196108 (2012).
2. Knirsch, K. C. *et al.*, "Mono- and ditopic bisfunctionalization of graphene", *Angew. Chem. Int. Ed.* **55**, 5861-5864 (2016).
3. Vecera, P. *et al.* Solvent-driven electron trapping and mass transport in reduced graphites to access perfect graphene. *Nature Commun.* **7**, 12411-12418 (2016).
4. Cançado, L. G. *et al.* Quantifying defects in graphene via Raman spectroscopy at different excitation energies. *Nano Lett*. **11**, 3190-3196 (2011).
5. Lucchese, M. M. *et al.* Quantifying ion-induced defects and Raman relaxation length in graphene. *Carbon* **48**, 1592-1597 (2010).
6. Englert, J. M. *et al.* Scanning-Raman-microscopy for the statistical analysis of covalently functionalized graphene. *ACS Nano*. **7**, 5472-5482 (2013).
7. Li, J. *et al.* Click and patterned functionalization of graphene by Diels-Alder reaction. *J. Am. Chem. Soc.* **138**, 7448-7451 (2016).
8. Bian, S. *et al.* Covalently patterned graphene surfaces by a force-accelerated Diels-Alder reaction. *J. Am. Chem. Soc.* **135**, 9240-9243 (2013).
9. Liu, L. H. *et al.* A simple and scalable route to wafer-size patterned graphene. *J. Mater. Chem*. **20**, 5041-5046 (2010).
10. Sun, Z. Z. *et al.* Towards hybrid superlattices in graphene. *Nat. Commun.* **2**, 559-564 (2011).
11. Syrgiannis, Z. *et al.* Reductive retrofunctionalization of single-walled carbon nanotubes. *Angew. Chem. Int. Ed,* **49**, *3322-3325* (2010).
12. Kessinger, R. *et al.* Preparation of Enantiomerically pure C_{76} with a general electrochemical method for the

removal of di(alkoxycarbonyl)methano bridges from methanofullerenes: the retro-Bingel reaction. *Angew. Chem. Int. Ed,* **37**, *1919-1922* (1998).

13. Moonen, N. N. P., *et al.* The chemical retro-Bingel reaction: selective removal of bis(alkoxyarbonyl)methano addends from C_{60} and C_{70} with amalgamated magnesium. *Chem. Commun.*, *335-336* (2000).
